# Patients’ and clinicians’ perspectives towards primary care consultations for shoulder pain: qualitative findings from the Prognostic and Diagnostic Assessment of the Shoulder (PANDA-S) programme

**DOI:** 10.1186/s12891-022-06059-1

**Published:** 2023-01-02

**Authors:** B. Saunders, C. Burton, D. A. van der Windt, H. Myers, R. Chester, T. Pincus, G. Wynne-Jones

**Affiliations:** 1 Primary Care Centre Versus Arthritis, School of Medicine, Keele University, Keele, Staffordshire ST5 5BG UK; 2 Clinical Trials Unit, Keele University, Keele, UK; 3 School of Health Sciences, Faculty of Medicine and Health, University of East Anglia, Norwich Research Park, Norwich, UK; 4 Faculty of Environmental and Life Sciences (FELS), University of Southampton, Southampton, UK

**Keywords:** Shoulder pain, Primary care, General practice, Diagnosis, Prognosis, Qualitative

## Abstract

**Background:**

Clinical management of musculoskeletal shoulder pain can be challenging due to diagnostic uncertainty, variable prognosis and limited evidence for long-term treatment benefits. The UK-based PANDA-S programme (Prognostic And Diagnostic Assessment of the Shoulder) is investigating short and long-term shoulder pain outcomes. This paper reports linked qualitative research exploring patients’ and clinicians’ views towards primary care consultations for shoulder pain.

**Methods:**

Semi-structured interviews were conducted with 24 patients and 15 primary care clinicians. Twenty-two interviews (11 patients, 11 clinicians) were conducted as matched patient-clinician ‘dyads’. Data were analysed thematically.

**Results:**

Clinicians reported attempts to involve patients in management decisions; however, there was variation in whether patients preferred treatment choice, or for decisions to be clinician-led. Some patients felt uncertain about the decisions made, due to a lack of discussion about available management options. Many General Practitioners expressed a lack of confidence in diagnosing the underlying cause of shoulder pain. Patients reported either not being given a diagnosis, or receiving different diagnoses from different professionals, resulting in confusion. Whilst clinicians reported routinely discussing prognosis of shoulder pain, patients reported that prognosis was not raised. Patients also expressed concern that their shoulder pain could be caused by serious pathology; however, clinicians felt that this was not a common concern for patients.

**Conclusions:**

Findings showed disparities between patients’ and clinicians’ views towards shoulder pain consultations, indicating a need for improved patient-clinician communication. Findings will inform the design of an intervention to support treatment and referral decisions for shoulder pain that will be tested in a randomised controlled trial.

**Supplementary Information:**

The online version contains supplementary material available at 10.1186/s12891-022-06059-1.

## Background

Musculoskeletal (MSK) shoulder pain is common and associated with impacts for the individual, healthcare and society [1, 2]. Many shoulder pain problems are managed predominantly in primary care, and in the UK, approximately 3% of adults consult their general practitioner (GP) for shoulder pain annually [3]. Whilst many shoulder pain problems are self-limiting and of short duration, 40–50% of people with shoulder pain continue to experience problems 6 to 12 months after first consulting their GP or physiotherapist [4, 5]. Individuals with persistent shoulder pain have reported negative impacts on daily activities including work, sleep, and self-care, e.g. dressing and bathing, as well as emotional distress and low mood as a result of shoulder pain [6, 7].

There are many uncertainties regarding the clinical management of shoulder pain. Whilst research has highlighted modest short-term effects of commonly used treatments such as corticosteroid injection, therapeutic exercise and manual therapy, there is limited evidence for long-term benefits [8–10]. There is a degree of uncertainty in relation to shoulder pain prognosis and diagnosis. Shoulder pain prognosis is variable, and systematic reviews of shoulder physical examination tests have highlighted variation in performance and interpretation of these tests, resulting in low diagnostic accuracy. There has also been found to be a poor correlation between diagnostic imaging findings, such as USS (Ultrasound Scanning) or MRI (Magnetic Resonance Imaging), and shoulder symptoms [11]. Despite this evidence, patients in Australia with rotator-cuff related shoulder pain were found to express the view that imaging is necessary for diagnosis of their shoulder pain [12]; and patients in Ireland with long-term shoulder pain were found to place importance on understanding the biomechanical cause of their pain [13]. It may therefore follow that uncertainties in relation to prognosis and diagnosis of shoulder pain could lead to patients having unmet expectations.

These uncertainties can also result in challenges for clinicians when deciding on the most appropriate treatments for patients. These challenges have been highlighted by general practitioners (GPs) in the Netherlands, particularly in relation to diagnostic uncertainty [14], and UK GPs have reported a lack of confidence in managing MSK problems in general, including shoulder pain [15].

Patients’ and clinicians’ views towards treatment decision-making have been explored in patients with long-term pain (the majority with over 1-year pain duration) [13]. However, the impact that diagnostic and prognostic uncertainties and challenges have on decision-making between primary care clinicians and patients at the level of individual consultations has not been fully explored in the literature. Gaining an in-depth understanding of primary care clinicians’ and patients’ perspectives is an important step towards identifying ways to better support clinicians and patients to address the current challenges and uncertainties in order to optimise treatment and referral decisions, which in turn may improve patient outcomes. The aim of this paper is therefore to address the following research question: what are patients’ and clinicians’ perspectives towards primary care consultations for shoulder pain? We explore influences on, and uncertainties regarding, treatment and management decisions for shoulder pain, including decisions about referral to other healthcare providers and/or referral for diagnostic imaging. Views on the importance of diagnosis and prognosis in guiding decision-making will be investigated.

This study is linked to an 8-year research programme funded by the UK National Institute for Health Research (NIHR) and Versus Arthritis: Prognostic And Diagnostic Assessment of the Shoulder (PANDA-S). PANDA-S is investigating the short and long-term outcomes of shoulder pain, with the aim of informing the design of an intervention to support clinicians and patients to make optimal decisions regarding self-management, treatment and referral. A longitudinal cohort study is currently being undertaken of 492 patients with shoulder pain presenting to UK NHS general practices and physiotherapy services [16]. Participants invited to take part in the qualitative study were recruited from the cohort (see Recruitment and Sampling for details).

## Methods

In the qualitative study, one-to-one semi-structured interviews were carried out at a single time-point with patients and clinicians, with data analysed using thematic analysis and drawing on the constant comparison method, influenced by grounded theory [17]. Where possible, interviews were carried out as ‘dyads’, i.e. the patient and clinician were interviewed separately about the same consultation. This was to allow for direct comparison between patients’ and clinicians’ accounts of how decisions about treatments and referral were reached. However, in cases where it was not possible to arrange dyad interviews (e.g. where the clinician did not respond to an interview invitation), standalone interviews were carried out.

The study settings were general practices and physiotherapy services in the West Midlands, North West and Thames Valley/South Midlands regions of England. The study received ethical approval from the NRES Committee Yorkshire & The Humber - Sheffield, 16/10/2018, ref.: 18/YH/0346.

### Recruitment and sampling

Patients were recruited by invitation letter and then via phone, having consented to contact as part of their participation in the PANDA-S cohort study. Patients were purposively sampled from the baseline questionnaires completed in the cohort, to capture a range of characteristics; including, age, gender, pain duration, self-reported pain intensity and geographical location. Patients who agreed to take part in an interview were also asked for their permission to contact the clinician with whom they consulted for their shoulder pain, in order to invite them to a separate interview. Patients gave permission for this in all cases.

Clinicians were contacted via emailing their practice administrator or practice manager, followed by a telephone call or further email. In cases where patients could not remember the clinician’s name, the practice manager first identified which clinician the patient had consulted, and then put the research team in touch with the clinician.

Given that all of the patients interviewed had consulted with a GP ─ as well as one First Contact Practitioner (FCP) (FCPs are utilised in UK primary care as the first point-of-contact for MSK presentations) who was based in a general practice─ at a later stage in the study recruitment it was decided to invite MSK physiotherapists to standalone interviews (i.e. not as part of a dyad), to also explore their views and experiences. Whilst the physiotherapists were invited from services participating in the cohort study, they had not consulted with patients recruited to the qualitative study. The intention was to explore their views about management of shoulder pain more generally, both in treating patients who had self-referred to physiotherapy services and those patients referred to physiotherapy from general practice. Physiotherapists were contacted directly via email and invited to participate. All of the physiotherapists who were invited agreed to be interviewed.

### Data collection

Eleven patient interviews took place at participants’ homes; and 13 via telephone. Interviews lasted between 21 minutes and 53 minutes. Five clinician interviews took place at their practice/clinic, and 10 were via telephone. Clinician interviews lasted between 19 minutes and 38 minutes. All of the interviews conducted after the start of the COVID-19 pandemic (9 patients, 8 clinicians) were via telephone. All interviews were conducted by BS (male, PhD), an experienced qualitative researcher from a social science background. BS was not previously known to the participants.

All participants were given an information leaflet explaining the study prior to providing written informed consent (for in-person interviews) or audio-recorded informed consent (for telephone interviews) at the start of the interview. Interviews were audio-recorded. Field notes were not made during interviews as it was felt this could negatively impact upon the rapport between researcher and participants. Informed consent was reaffirmed verbally at the end of each interview.

Separate topic guides were used for patient and clinician interviews, covering a range of areas relevant to the study aims (see Supplementary files A, B, C). The PANDA-S Clinical Advisory Group (CAG) and Patient and Public Involvement and Engagement (PPIE) group, helped to develop these topic guides. The PPIE group consisted of 4 patients with experience of receiving care for shoulder pain. The CAG group included 11 clinicians: 7 physiotherapists, 3 GPs and 1 rheumatologist. Topic guides were used flexibly within interviews, allowing the interviewer to follow up on any unexpected findings. To aid recall during interviews, several clinicians referred to the medical record from the consultation being discussed. However, only the parts of the medical record directly related to the patient’s shoulder pain management were communicated to the interviewer. Early interview findings informed subsequent data collection, with the topic guides iteratively revised throughout the data collection process.

### Data analysis

Audio-recordings of interviews were transcribed and anonymised. Transcripts were checked for accuracy against the audio, but were not returned to participants for comment or correction so as to avoid overburdening. Data were analysed thematically, using the constant comparison method [17], looking for connections within and across interviews, and across codes, highlighting data consistencies and variations. Analysis was an iterative process and data collection continued until saturation was judged to have been reached across both the patient and clinician datasets separately. Saturation was defined as ‘informational redundancy’– the point at which additional data no longer offers new insights [18].

Anonymised transcripts were first systematically coded on a line-by-line basis by BS, with the aid of the software program Nvivo 12, in order to identify recurrent concepts inductively. Coding was at first largely descriptive, and later became more conceptual as interpretations of the data moved towards a higher level of theoretical abstraction. Coding was reflexive and recursive, with codes being revisited in light of the findings of subsequent data-collection. Analysis began with the patient data and then mapped the views and experiences of clinicians against that, to allow for comparison between the two datasets.

Impressions of the data based on early analysis were discussed among the research team, and with the PANDA-S programme’s independent steering committee. Transcripts chosen at random from 2 patient-clinician dyads, i.e. 2 patient transcripts and 2 clinician transcripts, were then independently coded by two other members of the team. Coders brought different disciplinary perspectives to the data (BS: social science; GWJ: nursing, epidemiology; CB: general practice). The independent coding was carried out after the data collection had been completed. The aim was to understand cross-disciplinary perspectives on the data and, through discussion, to come to an agreement on shared meanings and interpretations. Based on these analyses, four main themes were developed. These themes, along with accompanying data extracts, were then presented to the study’s CAG and PPIE groups in separate meetings, in order to gain their perspectives. Members of both groups expressed strong agreement with the themes developed and the research team’s interpretations of these themes. Supplementary files D, E, F, G show a visual representation of the development of each theme, in the form of coding trees.

A further stage of analysis was then carried out in which identified themes were explored specifically in relation to the 11 patient-clinician dyads. This involved close analysis of each of the matched patient-GP transcripts, and the matched patient-FCP transcripts, in order to explore the key themes within the context of views towards individual consultations. Analysis was therefore both across and within cases, allowing for a broader understanding of patients’ and clinicians’ views, as well as fine-grained, idiographic investigation of patients’ and clinicians’ perspectives on individual consultations.

In what follows we outline the characteristics of the participant sample, before reporting the key themes.

## Results

Twenty-four interviews were conducted with patients and 15 with clinicians, between May 2019 and June 2021, which included a 10-month break in recruitment to the qualitative study from March 2020 to January 2021 as a result of the COVID-19 pandemic. Twenty-two interviews (11 patients, 11 clinicians) were conducted as ‘dyads’, where the patient and the clinician they had consulted with for their shoulder pain were interviewed separately about the same consultation. The other 17 interviews (13 patients, 4 clinicians) were standalone interviews, i.e. they were not part of a ‘dyad’. In the case of the 13 patient standalone interviews, this was because 13 of the ‘matched’ clinicians either did not reply to the invitation, or declined citing lack of time, and 11 were interviewed (resulting in 11 ‘dyads’).

The patients interviewed had all consulted with a GP, except for one patient who had consulted with an FCP. Patient and GP interviews were conducted as near as possible to the time of the consultation in which the patient was identified for PANDA-S. In some cases, this was several weeks, but others were interviewed a few months after the consultation. Thirteen patients had had one GP consultation at the time they were interviewed, in which they were first told about the PANDA-S study. Eleven reported in the interview that they also had consulted a second time, either due to ongoing symptoms, arranged follow-up, or to receive investigation results.

### Patient characteristics

Eleven patients were female and 13 male, aged from 38 to 79 years (average age: 62). Eleven patients were currently in employment, representing a range of occupation types, 12 were retired, and 1 was currently not in work. Patients were located across each of the UK geographical regions recruited from for the cohort: West Midlands, North West, South East and South Midlands. Pain severity at its worst over the past week, measured at baseline in the cohort study varied between 3/10 and 10/10, with an average of 6.7/10. Duration of the current episode of shoulder pain measured at baseline ranged from 2 to 6 weeks to over > 1 year. Table 1, below, summarises the characteristics of the 24 patients interviewed.

**Table 1:** Characteristics of the patients interviewed

Gender	Age	Geographical location within the UK	Duration of current pain episode	Pain severity at its worst over the past week (on a 0–10 scale, where 0 = no pain; 10 = worst pain)	Employment status	Job type	Work absence in previous month	Response to: how often do you need someone to help when you read written material from your doctor or pharmacy?
F	58	West Midlands	> 1 year	6/10	Not working due to ill-health	n/a	n/a	Rarely
F	79	West Midlands	2–6 weeks	3	Retired	n/a	n/a	Never
M	68	West Midlands	2–6 weeks	8	Retired	n/a	n/a	Rarely
M	74	South Midlands	> 1 year	8	Retired	n/a	n/a	Rarely
M	74	North West	6–12 weeks	4	Retired	n/a	n/a	Never
M	58	South Midlands	2–6 weeks	7	Employed	Not disclosed	No	Rarely
M	46	North West	3–6 months	6	Employed	Engineer	No	Never
M	60	North West	6–12 weeks	5	Retired	n/a	n/a	Never
F	67	West Midlands	6–12 weeks	8	Retired	n/a	n/a	Never
F	68	West Midlands	> 1 year	10	Retired	n/a	n/a	Not disclosed
F	55	South Midlands	6–12 weeks	10	Employed	Manager	No	Rarely
M	70	South Midlands	6–12 weeks	7	Retired	n/a	n/a	Always
M	69	West Midlands	2–6 weeks	10	Retired	n/a	n/a	Never
F	38	West Midlands	6 m-1 year	7	Employed	Cleaner	No	Never
M	57	North West	3–6 months	8	Employed	Technician	No	Never
M	53	West Midlands	6 m-1 year	8	Employed	Labourer	10 days	Never
F	39	South East	> 1 year	7	Employed	Instructor	No	Never
M	65	South East	> 1 year	10	Employed	Driver	No	Sometimes
M	70	South East	> 1 year	8	Employed	Not disclosed	No	Never
F	64	South East	6–12 weeks	6	Employed	Manager	No	Never
M	55	South East	6–12 weeks	7	Not working due to ill-health	n/a	n/a	Never
F	79	South East	> 1 year	8	Retired	n/a	n/a	Rarely
F	73	South East	2–6 weeks	8	Retired	n/a	n/a	Never
F	41	South East	6–12 weeks	5	Employed	Publisher	No	Never

### Clinician characteristics

Of the 15 clinicians interviewed, 10 were GPs, 1 was an advanced practice physiotherapist working in a general practice as a First Contact Practitioner (FCP), and 4 were physiotherapists. Eight clinicians were female and 7 male. Five GPs were female and 5 male; 2 MSK physiotherapists were female and 2 male, and the 1 FCP was female. The length of time clinicians had been practising ranged from 3 to 32 years. Clinicians were located across each of the geographical regions recruited from for the cohort.

### Main themes

The four main themes identified were:

1. Decision-making and discussion of management options
2. Diagnosis of shoulder conditions
3. Discussion of prognosis
4. Giving and receiving reassurance

These four main themes are outlined in the following sections. Supplementary file H describes each patient-clinician dyad interview, in turn.

#### Theme 1: decision-making and discussion of management options

Clinicians highlighted a number of considerations in the management of shoulder pain, such as the patient’s age, occupation, pain history and the impact of pain on their daily lives. However, they indicated that whilst these factors may influence their decisions, they routinely attempt to involve patients in decision-making through offering them a choice of treatments:

*You need to take into account the patient demographics: age, sex, occupation and their presenting history, history of how the pain has started and presents … but I think it’s got to be a shared decision process between you and the patient as to which route they want to take … ultimately it’s patient choice. (Physiotherapist, male, 8 years practising)*

This correlates with the views of some patients who reported making the final decision between treatments offered to them by the clinician:

*P: He said I could leave it a while and have an injection, or I could refer you to physiotherapy. So, I thought I’d try physio*

*Int: So it sounds like the doctor gave you some choice of which you preferred?*

*P: Yeah, it was my choice … I thought the physio might be a better long-term solution to the underlying problem rather than injection (Female patient, aged 64)*

Clinicians reported that, in the absence of ‘red flag’ symptoms, in most cases they favoured initial conservative management, such as self-management advice, analgesia or referral to physiotherapy. However, some clinicians reported offering more invasive options, such as corticosteroid injection, at an early stage if the pain was significantly impacting the patient’s everyday life, or the patient was seeking short-term pain relief. This was the case in the following extract from a clinician-patient dyad, in which the GP gave the patient an injection in the consultation:

*So there was bicipital tendinosis, and [the patient] came back (for a follow-up consultation) and said the pain was still the same, it was still disturbing his sleep, what would be a quick fix. So we spoke about the pros and cons of injection, and he did have an injection … people do come with a pain, it’s affecting their sleep, it’s affecting their lifestyle. For people like this patient, who it does give them relief, then I think it’s a service that’s appreciated. (GP, female, 32 years practising)*

The matched interview in this dyad shows consistency with the clinician’s account, in that the patient also highlighted the impact of the pain on his sleep as being his main concern. The patient reported having been hesitant about an injection, but expressed a strong preference for the decision about his treatment to be led by the GP:

*It was a few days after when it started to occur and I couldn’t sleep. Oh, it was a nightmare. The whole of my arm ached … I couldn’t care less about medical stuff. I’ll leave that to the doctors. I’m not interested. All I want them to do is to treat me. So all I wanted, get rid of this pain in my arm, whatever way, let them tell me. I didn’t fancy an injection in my shoulder. I got home and thought, ‘What have I done?’. But do you know, from then on after that night it went easier. (Male patient, aged 64)*

Clinicians similarly highlighted this preference of some patients not to be involved in their treatment decisions and instead wanting the clinician to take a directive approach, deferring to their clinical knowledge:

*I do try and do shared management decision-making as much as possible with people and some patients are very amenable to that, and other people say ‘Well you’re the doctor, what should I do? What would you recommend?’. And then you think ‘Right okay, I think we probably should do this first and this next’. (Female GP, 3 years practising)*

However, some patients’ views challenged the dichotomy between patient choice and clinician-led decision-making. They indicated that, even if they preferred the eventual decision to be taken by the clinician, they still wanted to engage in a form of shared decision-making involving discussion about available options and for the clinician to outline a potential treatment pathway. However, for many patients they reported that these discussions had not taken place:

*He basically told me what it was [i.e. bursitis], felt along my shoulder and explained where things were and that there was the fluid sac or something, so basically that was it … if other treatment is available like physio or if I need another scan or anything like that, I would have liked to have discussed that because I don’t just want to be told, ‘oh well it’s this, learn to live with it’. (Female patient, aged 38)*

Some patients also reported a lack of information from the clinician about why the recommended treatment was the most suitable and how it would treat their symptoms. They seemed to indicate a reluctance to raise these concerns during the consultation, but then felt dissatisfied following the consultation:

*Basically the doctor asked me, would I like to have the steroid injections and I asked him ‘would it do me any good?’; which he said he thought it would, made my appointment … and that was basically the end of the consultation … when you come away you think, ‘what did I learn then?’. It was nothing, I learnt nothing, I didn’t know what the injection would do or why. (Male patient, aged 70)*

#### Theme 2: diagnosis of shoulder conditions

Receiving a diagnosis for their shoulder pain was important to patients, partly to reassure them that the pain was not caused by serious pathology (this is explored further in the ‘Giving and receiving reassurance’ theme, later), but also this was seen to be key to receiving the right management:

*It’s nice to be told what it is [i.e. bursitis] … they could actually tell me what I can take to relieve the pain and not just keep saying ‘oh we don’t know, try this, it’s just something you are going to have to live with’ … I know now that if it gets worse I can go to the doctors and they are aware of what it is and maybe how they can treat it. (Female patient, aged 38)*

However, several patients reported that their GP either did not offer them a diagnosis or explanation of possible causes of their shoulder pain, or that they conveyed uncertainty. In many cases this resulted in the patient being referred for further investigation:

*The GP thought that it was potentially something to do with the ligaments, just something’s worn out there and she thinks the bones are actually scratching on each other and that’s why it’s causing this sharp pain. It’s like the nerve’s been damaged there, that’s what she thinks has been going on … she will see on the ultrasound. She can’t say 100% but that’s what she's guessing. (Female patient, aged 39)*

This diagnostic uncertainty was reflected in clinicians’ views. Many GPs reported lacking confidence in diagnosing shoulder conditions due to a perceived lack of skill in shoulder examination, leading them to commonly send patients for investigations such as an ultrasound scan:

*I’m conscious that I do probably far too many ultrasounds … and I probably need to brush up on specific shoulder examination skills in order to be able to more specifically diagnose a specific tendonitis or bursitis or impingement or whatever, rather than just thinking ‘ooh it could be this that or the other’, without having to image; unless there were any red flags obviously. (GP, female. 32 years practising)*

There was variation in the data, however, as all of the physiotherapists and a few GPs reported having greater confidence in diagnosing shoulder conditions. For many of these clinicians, providing a diagnosis was perceived to be a key part of their professional role:

*If my role isn’t to diagnose people, then what am I doing? Of course it is … I’m perfectly happy in that part of my role. But I also am aware sadly of things like NICE [National Institute for Health and Care Excellence] guidance which seems a lot more investigation-focused, and I do think maybe some of my younger colleagues are more inclined to sort of hedge before making a definitive diagnosis, whereas I’m quite happy to put the code in, subacromial and move on. So yes, I think that’s an absolutely crucial part of my role*. *(GP, male, 28 years practising)*

The majority of clinicians reported that during the initial stages of the COVID-19 pandemic, the need to conduct consultations remotely via telephone or video call had made diagnosing shoulder conditions more difficult. This was because in many cases physical contact with the patient was felt necessary for assessment and diagnosis. Additionally, examinations were limited by the patient’s ability to follow the clinician’s instructions in carrying out arm movements, as well as ensuring that these movements were captured on the screen:

*To assess strength appropriately in different movements and different directions, you’re reliant on the patient having something suitable in their home to be able to do it for starters. Secondly, then to be able to position the phone in such a way that you can see them doing it, which sometimes isn’t the case. You also then need to be able to guide them with the technique … one of the things then you’re looking at with your rehab folk is trying to normalise that movement pattern. Sometimes that does require physical feedback, not just verbal feedback. So I think it’s a challenge to do over video. It’s not impossible, but it’s immensely difficult. (Physiotherapist, male, 11 years practising)*

This view was mirrored by patients, who felt that remote examinations were less thorough than in-person, hands-on examination, and also reported difficulty performing movements in line with the clinician’s instructions over video. As a result, patients reported feeling less confident when a diagnosis was provided:

*You don’t get a full examination unless you meet the person I’d imagine. I think you can only do so much on the telephone or on video. I’m disappointed really that she was taking my [previous] diagnosis without examining me … I’ve had a video call from her and it was comical, absolutely comical. ‘Right put your phone in front of you, lift your arm up so I can see it’. [I replied] ‘Look, if I put my phone there I can’t see it, so I can’t see what you can see, so I can’t do the exercise’. (Male patient, aged 53)*

Several patients reported receiving different diagnoses from different professionals, leading them to experience confusion. This is exemplified in the following clinician-patient dyad. The patient reported feelings of uncertainty, having been told by his GP that he had arthritis, but by another professional that he had subacromial shoulder pain (i.e. impingement):

*They’ve been telling me different things. The first doctor who rang said it’s just arthritis … they referred me to physiotherapy … but the man who did my scan at the hospital told me it was quite a bad impingement. So I didn’t know which one to believe. (Male patient, aged 65)*

The First Contact Practitioner (FCP) that the patient consulted with explained that, based on the patient’s X-ray and her examination, both diagnoses were indicated:

*He had an X-ray of his shoulder which showed calcific rotator cuff tendonitis and subacromial bursitis and then the ultrasound showed some moderate OA (osteoarthritis) changes in his acromioclavicular joint. Normal long head of biceps, no rotator cuff tear but some thickening of the bursa which was felt likely to be representative of impingement. (FCP, female, 24 years practising)*

#### Theme 3: discussion of prognosis

Patients reported concerns about how long their shoulder pain might last and whether it could worsen or recur. In some cases, patients appeared to catastrophize about the future:

*The pain is getting worse and there will be a point where I won’t be able to move my arm beyond that point [signals waist height]. And so that’s obviously going to limit my movement, which will impact my job severely. And who knows?” (Male patient, aged 46)*

However, the majority of patients reported that their shoulder pain prognosis had not been discussed with the clinician. Many perceived that this was because the clinician was unlikely to know how their pain may progress, particularly if a diagnosis had not been established. Some patients appeared accepting of this uncertainty so long as a clear management plan was in place:

*Int: Did the GP give you any idea about the future, whether it might be something that could be resolved, or … ?*

*P: No, no, nothing, the GP didn’t really say, but nobody knows … she doesn’t really know exactly what's wrong with me, she can’t give me promises, because then I could be, ‘oh yeah, she said it’ll be fine, you know after this injection’, and then if it isn’t, I’d be like well ‘why did she say that?’ So I think it’s more not giving me false hope, she's like ‘okay, let’s see what we can do, we can offer you this and that, and if none of this works, we will try something else’. (Female patient, aged 49)*

In contrast to this, other patients reported that they would have liked their prognosis to have been discussed in the consultation:

*Int: Did they talk to you at all about the future outlook, the prognosis?*

*P: No, nothing of the sort. No, we didn’t talk about that*

*Int: Is that something you’d liked to have discussed with the clinician?*

*P: Absolutely yeah. Managing expectations yeah, it’s a big thing isn’t it? Just to give me an idea if you’re going to get better or have I just got to live with it. (Male patient, aged 53)*

Clinicians’ accounts showed discrepancy with these views, as all clinicians reported that they routinely discuss shoulder pain prognosis in consultations. They perceived this to be important for encouraging patients to engage with a management plan:

*Int: Would you discuss with patients how their pain might progress?*

*P: Yeah all the time. I usually say, ‘right, for this sort of problem, from previous experiences and from the research out there it looks like it will take X amount of time to get better and if you engage with your physio programme the aim is to reduce that timescale of course’ … just give them that advice on timescales and what we do next should they not improve within that timescale. (Physiotherapist, female, 6 years practising)*

Some clinicians reported a more cautious approach whereby they give patients a general idea of a timeframe for recovery rather than a specific prognosis. They also highlighted the importance of ensuring patients have realistic expectations about their recovery:

*I want people to be realistic, so I don’t want them to expect it to get hugely better immediately and then be upset that it isn’t. So I will often say something like, ‘Shoulder problems can go on for months’ because I want them to have some realistic expectations. But I would then try and counter it by saying, ‘But if we do this and that and the other we’ll aim to make it feel better along the way and improve more quickly.’...because if you tell someone with a frozen shoulder the natural history is 18 months without any treatment, I mean that’s just depressing, but also realistic. So I kind of hedge my bets in the middle a little … what I don’t want them to do is ring me in a week and say, ‘It’s not better yet.’ So it’s just getting that balance really. (GP, female, 15 years practising)*

The disparity in patients’ and clinicians’ accounts of discussing prognosis in consultations is exemplified in patient-clinician dyads. The following patient reported that neither diagnosis nor prognosis were discussed with his GP, and that his expectation was that his shoulder pain may never resolve:

*The doctor didn’t actually say what he thought it was, he just thought the way to go forward was physiotherapy … my feeling is that I’ve probably got to live with it. I don’t really know what to expect. I’m not optimistic because I’m not 100% sure it’ll ever go. (Male patient, aged 74)*

This contrasts with the GP’s account in which he reports having communicated to the patient a diagnosis of frozen shoulder and outlined the likely timeframe for recovery:

*Int: Did you discuss [the pain condition] with him in the consultation?*

*P: Yes, yes. I always do, I always give explanation to patients that what I think it is, in lay terms, a frozen shoulder, and I gave him an idea of what it was about and then I referred him to physio … prognosis is vitally important. You need to give them an idea of timeframe because otherwise they’re not getting better. You’ve got to tell them the likelihood, how long it’s going to last before getting better, otherwise they come back and they don’t have faith in your management. (Male GP, 34 years practising)*

#### Theme 4: giving and receiving reassurance

Clinicians reported that patients commonly needed reassurance about the impact of shoulder pain on their function and daily activities. However, they felt that, unlike spinal pain, patients do not tend to have concerns about serious pathology related to their shoulder:

*I don’t see necessarily that patients have that mentality and worrying about it being something nasty. However, I do see a lot more that they worry about whether they’re allowed to do certain things. So are they allowed to lift their arm if it’s painful? So I see a lot more worry about the pain or how long did it last rather than it being due to a non-MSK or a more sinister pathology. (Physiotherapist, male, 11 years practising)*

Patients’ accounts showed some discrepancy with these views, however, as patients reported concerns that something serious may be causing their shoulder pain, such as cancer, or a serious injury to the bones or ligaments. They therefore reported feeling reassured if they received a diagnosis:

*Looking for exercises to help it, you go onto Google and it comes back you’ve got cancer straight away, isn’t it? Everything, ‘Oh yeah, points to cancer’ … Once I knew it was tendonitis I was happy. I wasn’t happy, but I was happy if that makes sense*. *(Male patient, aged 46)*

The disparity between clinicians’ and patients’ views is reflected in the following dyad. The patient expressed concern that something serious may be wrong with his shoulder, but this appears to have either not been communicated, or not identified by the clinician:

*You always think these things, is it some sort of bone cancer? Or, I’m not sure what can be done on ligaments or muscles that would be more serious, I’m not that familiar with things that go wrong in your shoulder. But yes you always wonder could it be something very serious, have I done some serious injury and not realised it? Is it some disease that’s eating away at my muscle or bone? (Male patient, aged 60)*

*I don’t think it was anxiety as such. It was more like he said, ‘I’d like not to have this pain please, if you can?’ rather than anything else. I certainly wouldn’t describe anxiety as a presenting feature with this chap. (GP, male, 24 years practising)*

In some cases, when asked in interviews patients reported that they had not in fact expressed these concerns to their clinician:

*P: When you get things like this you think the worst. I’m a bit of a worrier and I just think oh, cancer or something like that*

*Int: Was she able to reassure you that it wasn’t?*

*P: No, no, no*

*Int: Did you express those concerns to her?*

*P: Well I didn’t, no. I didn’t actually, no. (Female patient, aged 56)*

Some patients who had not received a diagnosis for their shoulder pain suggested a tension whereby on the one hand they felt a sense of reassurance through having confidence in the expertise of their clinician, particularly where there was a strong therapeutic rapport, but at the same time they felt a lack of reassurance with regard to the cause of their pain:

*She allows you time to go through everything and talks to you about everything, she’s a fabulous doctor. But in this situation she wouldn’t be able to tell me exactly what it was … so it was sort of reassuring but it wasn’t, at the same time. (Female patient, aged 68)*

A few GPs displayed a recognition of this tension, and highlighted the challenge they faced in attempting to reassure patients within the frame of diagnostic uncertainty. They reported addressing this through being honest about these uncertainties, and involving the patient in discussing management:

*I think [the patient] was happy that the X-ray didn’t show any arthritis particularly, but I think he was then worried to say ‘Well what is it? What’s causing it?’. And I think that’s the difficult point where you say ‘Well it could be any number of things’ … I believe very much so that we need to be honest with our patients if we don’t know the answer. What I often will say is ‘Look, these are the possibilities and these are the things I’m going to do to rule in or rule out those possibilities’. And I try and have a conversation about it and do shared management decision-making as much as possible. (Female GP, 6 years practising)*

Clinicians also highlighted the need, in some instances, to provide reassurance about the relative value and risks of treatment options; for instance, reassuring patients that physiotherapy will not cause them harm or worsen their pain, even if the cause of pain is unclear:

*Sometimes patients are nervous to go to physio if they don’t know what’s going on and we do find that’s sometimes a bit of barrier. Some of these people find it very reassuring to have something in terms of a scan or an X-ray that says ‘This is what’s happening’. Some people seem to worry that the physio will make things worse and I try to explain to a lot of patients that physio is very unlikely to make anything worse; chances are it’ll probably make things better. (Male GP, 20 years practising)*

## Discussion

In this article we aimed to explore, and develop a better understanding of, patients’ and clinicians’ perspectives towards primary care consultations for shoulder pain. This aim was achieved through the generation of rich, in-depth insights regarding the management of shoulder pain, from the perspectives of the two groups of participants. In particular, the findings show some consistencies between the views of patients and primary care clinicians towards shoulder pain management, but also a number of disparities, which could reflect miscommunication or misunderstanding within consultations, or a lack of recall following the consultation. Both clinician and patient data indicated that there was variation in patients’ preferences towards shared decision-making. However, some patients reported leaving the consultation with a lack of clarity about their management, as they did not feel that available management options had been discussed, nor a clear treatment pathway outlined. Diagnostic uncertainty was apparent in the accounts of some clinicians and patients, and as a result patients expressed concerns that there may be something seriously wrong with their shoulder. Clinicians perceived these types of concerns to be uncommon for patients with shoulder pain, and therefore did not always provide the ‘cognitive reassurance’, i.e. information about aetiology [19] that patients sought. However, it appears that this may be due to patients’ reluctance to raise these concerns with their clinician. Some patients highlighted a tension between feeling reassured on the one hand due to the confidence and trust they have in their clinician, i.e. ‘affective reassurance ‘[19], yet at the same time feeling a lack of cognitive reassurance if they had not received a diagnosis to alleviate their concerns about serious pathology or injury. Discrepancy was also observed in that patients reported that their prognosis had not been discussed in their consultation, whereas clinicians reported that they routinely outlined a timeframe for recovery, which they perceived to be important for patient outcomes. It may be that this was either not clearly communicated, or not comprehended within consultations, or that patients and/or clinicians had difficulty reliably recalling these discussions.

### Comparison with other literature

Some of our findings show similarity with previous qualitative research on shoulder pain management. The importance that patients placed on receiving a diagnosis for informing the management of their shoulder pain corroborates Cuff and Littlewood’s [20] findings on patients with subacromial impingement syndrome. However, Cuff and Littlewood reported that patients in their study were able to give a clear and accurate explanation of their diagnosis, which differs from our finding that some patients reported confusion about the cause of their pain even when they had been given a diagnosis. This difference may be explained by Cuff and Littlewood having recruited patients referred for physiotherapy at an orthopaedic shoulder clinic, who may have received a more detailed explanation of their condition in this setting. Our findings perhaps align more with Ottenheijm et al.’s [14] study of Dutch GPs, who reported patients’ confusion about their shoulder pain diagnosis due to diagnostic disagreements between different professionals. We also observed patient confusion due to having received different diagnoses. However, this was not caused by cross-professional disagreement, but was due either to a lack of explanation to the patient that different diagnoses can co-occur, or patients not recalling that this was explained to them.

Another similarity between ours and Ottenheijm et al’s [14] findings relates to GPs’ uncertainty about shoulder diagnosis; some GPs in their study also felt that their diagnostic knowledge was insufficient in relation to the shoulder. This is corroborated by a UK-wide survey of 714 GPs which found that diagnostic uncertainty commonly resulted in GPs referring patients for further investigations such as blood tests, radiographs and USS [21]. This may help to explain why a number of patients in our study were referred for diagnostic tests such as X-ray and ultrasound. However, this finding may also reflect an importance being placed on establishing a diagnosis and a desire for a diagnostic label, which may suggest biomechanical beliefs about shoulder pain held by patients and some GPs. This aligns with Maxwell et al’s [13] findings that biomechanical beliefs about shoulder pain held by clinicians and patients with long-term shoulder pain in Ireland, affected decision-making about treatments, imaging and onward referrals.

Our finding that patients experienced concerns and anxiety about their shoulder pain shows similarity with other studies. However, the patient concerns about serious underlying pathology such as cancer, or serious injury, that we identified were not observed in these studies. Instead, patient anxiety was found to be a result of delays in receiving a diagnosis [22], functional limitations of shoulder pain [7], and fears about symptom exacerbation [6]. Similar to our study, a qualitative review by Cheung and Soundy [23] highlighted the importance to patients with shoulder pain that their worries and concerns are addressed through clinicians providing both affective and cognitive reassurance [19]. However, this review did not draw out the tension we observed that patients experience when one form of reassurance is provided but not the other, nor the challenges clinicians face in reassuring patients in the absence of a diagnostic explanation.

The issues and concerns identified among patients and clinicians about shoulder pain consultations being carried out remotely via telephone or video, show both similarities and differences with other literature. Donaghy et al. [24] found that GPs and patients responded positively to video consultations, generally finding them helpful and convenient. However, their study looked at primary care consultations more broadly, and not specifically shoulder pain. It was also carried out prior to the COVID-19 pandemic; therefore, at this time clinicians and patients would have had more choice as to which types of problems could be most effectively managed via remote consultations. A later, post-COVID-19 study by Murphy et al. [25], showed mixed findings, with GPs highlighting that for certain conditions there is a need to examine the patient in-person in order to visualise signs and symptoms close-up. This shows similarity with our findings, as both patients and clinicians indicated a strong preference for hands-on, in-person examination of the shoulder.

### Strengths and limitations

A strength of this study is the parallel investigation of the views of both patients with shoulder pain and primary care clinicians. This included 11 patient-clinician dyads (i.e. 22 matched interviews), which represents a novel aspect of the study, allowing for direct comparison of views towards the same consultation. The multi-disciplinary team involved in data analysis is also a strength, as well as the input from our PPIE group and Clinical Advisory Group, which increases the trustworthiness of the findings presented. A further strength is that whilst this study was conducted in a UK NHS setting, because of the focus on the primary care consultation itself, many of these findings could also be applicable to other non-UK settings.

A limitation of the study is that participants were interviewed several weeks after the consultation took place, therefore recall will likely have been a factor in some cases. GPs were able to use their clinical notes to aid their memories, but these notes would likely have included only a brief record of the decisions made. Therefore, where differences were identified between patients’ and clinicians’ accounts of what was discussed in the consultation, this disparity could have been due, in part, to participants misremembering, or being unable to recall certain details. However, this does not negate the usefulness of the findings, because if patients were unable to recall crucial information about diagnosis, prognosis or treatment decisions, or if clinicians recorded or recalled information differently from the way in which it was discussed, this can represent a key barrier to positive patient experiences of care and possibly outcomes.

Another potential limitation is the possibility of selection bias in the clinicians who participated. Thirteen of the clinicians ‘matched’ to patients either did not reply to the invitation, or declined citing lack of time; therefore, those who did agree to be interviewed may have differed in some ways. For instance, it may be that the clinicians who agreed to be interviewed had more interest or experience in managing shoulder pain, or more interest in research generally, and therefore were more motivated to participate. Whilst we do not have the information available to assess for any differences in characteristics between those clinicians who did and did not participate, this potential for difference is something that should be considered when interpreting the interview findings.

When interpreting the findings, it is important to acknowledge the influence of the researcher on participants’ responses in interviews; however, a reflexive approach was adopted throughout, in which the researcher attended to, and acknowledged any biases and preconceptions. Patients and clinicians were made aware that the researcher conducting interviews (BS) was a social scientist and was not from a clinical background, and that the team were interested in understanding both positive and negative aspects of their experiences. The other team members involved in the analysis were from nursing (GWJ) and general practice (CB) backgrounds, and these clinical backgrounds will likely have influenced their interpretations of the data. CB, in particular, currently works as a GP alongside her academic role, and therefore brought a general practice perspective to the data. This may have resulted in preconceptions about primary care shoulder pain management that were informed by her own management in consultations, but this may also have been of benefit in interpreting the clinicians’ reported views and experiences.

## Conclusions and implications

In this paper we have presented findings on patients’ and clinicians’ views towards primary care consultations for shoulder pain. This included, influences on, and uncertainty regarding, decision-making and the importance of diagnostic and prognostic information when negotiating treatment and referral options.

There are a number of implications that can be drawn out for future clinical practice and research. This is particularly in relation to the disparities identified between patients’ and clinicians’ accounts of the consultation and its outcomes. These disparities point to apparent miscommunication and/or miscomprehension and lack of recall of key information following the consultation. This may have led to many patients reporting uncertainty about their shoulder pain diagnosis, prognosis or the available treatment options. It is therefore important that, in line with UK National Institute for Health and Care Excellence (NICE) guidelines for shoulder pain management [26], clinicians should consistently provide clear information about diagnosis and prognosis, including any uncertainties, as well as explicitly checking patients’ comprehension of this information. There is also a need to make patients aware of the possibility that their pain may have more than one cause; for instance, arthritis may be present alongside other diagnoses such as subacromial shoulder pain. This can avoid any patient confusion in receiving more than one diagnostic label. Linked to this is the importance of fostering better cross-disciplinary communication regarding the information patients are given about their diagnosis and prognosis, to minimise the chance of patients receiving what they perceive to be conflicting information. Future research could build on these findings through video/audio recording or directly observing shoulder pain consultations and adopting a conversation- or discourse analysis approach to tease out the intricacies of the miscommunication and interactional difficulties identified in the findings.

With regard to decision-making about treatment and referral options, training for clinicians should address the nuances around shared decision-making for shoulder pain. Whilst clinicians were aware of the importance of shared decision-making, this appeared to be dichotomised in terms of patients either wanting to be offered a choice of treatments such as physiotherapy or corticosteroid injection, or preferring the decision to be clinician-led. Clinicians should therefore be aware that even when the patient displays an apparent lack of willingness to engage with shared decision-making focused on patient choice, they may still wish to engage in a different form of shared decision-making. Clinicians should therefore be encouraged to discuss all of the available options with patients and outline a clear treatment pathway. Additionally, patients’ worries about their shoulder pain being related to serious underlying pathologies such as cancer were not identified by clinicians, perhaps due to patients’ reluctance to voice these concerns to their clinician. It is therefore important that clinicians are made aware of the need to always ask patients If they have any specific concerns about their shoulder pain, in line with the ICE consultation model (ideas, concerns, expectations) [27]. They should also look to provide cognitive reassurance to patients, in the form of reassurance about the absence of any indicators of serious pathology, even if these concerns are not explicitly raised by the patient.

The findings presented here will directly inform the design of a clinical intervention as part of the PANDA-S programme, that will be tested in a future RCT. The intervention will address the issues raised in these findings, with the intention of helping clinicians to identify and address the needs, concerns and priorities of patients with shoulder pain, and to improve communication about treatment and referral decisions. Through this we will aim to optimise the management of shoulder pain within primary care consultations, with the goal of improving patient outcomes.

## Supplementary Information

**Supplementary file A.** PANDA-S Interview Topic Guide: Patients.

**Supplementary file B.** PANDA-S Interview Topic Guide: Clinicians - GPs.

**Supplementary file C.** PANDA-S Interview Topic Guide: Clinicians - Physiotherapists.

**Supplementary file D.** Theme 1.

**Supplementary file E.** Theme 2.

**Supplementary file F.** Theme 3.

**Supplementary file G.** Theme 4.

**Supplementary file H.** Outline of patient-clinician dyad interviews.

## Data Availability

In line with the Standard Operating Procedures in place at Keele School of Medicine, where this study was conducted, data are archived at a dedicated location within the Keele University’s network. A request to access archived data can be made by completion of a Data Transfer Request form, which can be accessed by contacting: Primary Care Centre Versus Arthritis, School of Medicine, Keele University, Staffordshire, ST5 5BG, UK; Tel: + 44 (0) 1782 733905.

## Abbreviations

GP: General Practitioner/ General Practice
RCT: Randomised Controlled Trial
NHS: National Health Service
PANDA-S: Prognostic and Diagnostic Assessment of the Shoulder
