# Supplementary material for: Patients’ and clinicians’ perspectives towards primary care consultations for shoulder pain: qualitative findings from the Prognostic and Diagnostic Assessment of the Shoulder (PANDA-S) programme
Source: BMC Musculoskelet Disord. 2023 Jan 2;24:1. doi: 10.1186/s12891-022-06059-1 (PMC9805906; doi:10.1186/s12891-022-06059-1)
Supplement: Supplementary file 1 — Supplementary file A. PANDA-S Interview Topic Guide: Patients. [file 12891_2022_6059_MOESM1_ESM.docx]

PANDA-S Interview Topic Guide: Patients

# Introduction

- 1. Check that participant has read and understood the PIS.
  2. Explain arrangements for: consent, recording, anonymity, expenses where appropriate etc.
  3. Check patient’s recollection of shoulder pain consultation (this will have been asked initially when arranging the interview)

# Experiences of shoulder pain (where possible invite participants to expand on responses)

- 1. Can you tell me about your experiences of managing your shoulder pain condition?
  2. What do you think caused your shoulder pain?
  3. In what ways has it affected your life?

Prompt on issues such as:

- - 1. everyday activities
    2. employment or unpaid work
    3. social/ family relationships
    4. impact on mood
    5. coping/ management strategies
  1. How (if at all) has the Covid 19 pandemic impacted on your experience of shoulder pain?
     1. Prompt re impact on shoulder pain self-management, i.e. has managing shoulder pain been affected by Covid? Any additional challenges or barriers?
     2. Impact of Covid on work? Family? If, so has this had any impact on experience of shoulder pain?
     3. Anxiety/ distress related to managing shoulder pain during Covid?

# Views on consultation for shoulder pain (where possible invite participants to expand on responses)

- 1. Consultation medium - was your consultation F2F, phone, video?
  2. If phone/ video, how did you feel about this?
     1. Prompt re perception of main differences compared to F2F
     2. Views on rapport building via phone/video.
     3. View on virtual examination (if applicable) – did this impact experience of consultation, reassurance about pain etc.?
     4. Perceptions about reassurance over the phone/via video
  3. For what reasons did you decide to go to your GP/ physio about your shoulder pain?
  4. Does your own view on the cause of pain match up with what you were told by your GP/ physio about possible causes?
  5. What were your expectations prior to the consultation, e.g. about tests, treatments, and what could be done for your pain?
     1. Did you have any particular treatment preferences?
     2. Were these expectations met?
  6. Have you been given a diagnosis or label for your condition?
     1. If so, what is your understanding of this diagnosis?
     2. Do you agree with this diagnosis?
        - If not, prompt as to reasons for this.
     3. How important is it for you to have a diagnosis?

- Prompt re issues such as gaining legitimacy, reassurance etc.

- 1. Were you given any diagnostic tests, physical examination or a scan?
     1. What are your views on the importance of these?
     2. To what extent were these tests/ examinations appropriate in your opinion?
  2. To what extent did you feel reassured about your shoulder pain as a result of the consultation?
     1. Prompt re reassurance about what, e.g. diagnosis/ cause of pain; ruling out pathology; reassurance about finding a cause/ diagnosis; reassurance i.e. treatment options?
  3. Did you feel confident in the knowledge and expertise of the GP/ physio who treated you?
  4. What did your GP/ physio tell you about how your pain is likely to progress and the likely outcome?

- Prompt about perceived helpfulness or reassurance of this prognostic info.

- 1. What advice were you given about managing your pain, work and other activities?
     1. How helpful was this information/advice?
     2. How confident do you now feel in your ability to manage your shoulder pain?
     3. Has the way you manage your shoulder pain changed as a result of your consultation, and if so, how?
  2. Have you been following this advice since the consultation?
     1. Prompt as to reasons for following/ not following clinician’s advice.
  3. To what extent did you feel involved in decisions about your treatment during the consultation?
  4. How do you feel about the relationship you were able to establish with your GP/ physio in the consultation?
     1. Did you feel listened to?
     2. How important is this relationship to you?

# Close of discussion

- 1. Any other final remarks/additional views.
  2. Check that consent is still in place.
  3. Reimbursement of travel expenses etc. (where appropriate).
