# Supplementary material for: Patients’ and clinicians’ perspectives towards primary care consultations for shoulder pain: qualitative findings from the Prognostic and Diagnostic Assessment of the Shoulder (PANDA-S) programme
Source: BMC Musculoskelet Disord. 2023 Jan 2;24:1. doi: 10.1186/s12891-022-06059-1 (PMC9805906; doi:10.1186/s12891-022-06059-1)
Supplement: Supplementary file 2 — Supplementary file B. PANDA-S Interview Topic Guide: Clinicians - GPs. [file 12891_2022_6059_MOESM2_ESM.docx]

PANDA-S Interview Topic Guide: Clinicians - GPs

# Introduction

- 1. Check that participant has read and understood the PIS.
  2. Explain arrangements for: consent, recording, anonymity, expenses where appropriate etc.
  3. Check clinician’s recall of specific patient and consultation being discussed; if necessary arrange to use consultation notes, patient history as an aid.
  4. Record the qualifications (level, experience), and if they have received specific MSK / shoulder training.

# Views and experiences of treating/managing shoulder pain

1. Mode of consultation – differences in consulting with shoulder pain patients remotely vs. F2F in current Covid context:
   - Suitability of remote consulting for shoulder pain
   - barriers/ challenges
   - what would prompt you to bring a patient in for a F2F consultation?
   - How do you see the use of remote consulting for shoulder pain going forward?
2. Can you talk me through your decision making in the management of this patient?
   - Were any tests/ examination/ imaging carried out with this patient?
   - Prompt clinician to discuss more generally the applicability of, and value attributed to, diagnostic tests, including physical examination and imaging.
   - How has decision-making re management been impacted by the Covid pandemic?
     - Has this changed how you’ve approached management decisions in any way? If so, how?
3. What factors influenced your treatment and referral decisions *(if applicable)* for this patient?
4. Differences in management based on patient characteristics:
   - Age – younger vs. older patients?
   - Those in work vs. retired patients?
   - Acute injury vs. longer term pain problems.
5. What are your views on reassurance for shoulder pain patients?

- Prompt to discuss specific consultation, but also more broadly.

- Does worry/ anxiety appear to have a big role for shoulder pain patients?

- Do you routinely explore issues re worry/anxiety related to Covid? Was this an issue for the specific patient?

1. How important do you regard making a diagnosis in the case of shoulder pain?
   - How would you define making a diagnosis in relation to shoulder pain?
   - How confident do you feel diagnosing shoulder conditions?
   - Are there differences for shoulder pain in terms of making a diagnosis when compared to MSK pain in other body site regions?
2. How important do you feel it is to communicate a likely prognosis to the patient?
   - What would be your definition of prognosis in relation to shoulder pain?
   - How confident do you feel in communicating a likely prognosis?
   - Are there differences for shoulder pain in terms of assessing prognosis when compared to MSK pain in other body site regions?
3. How did you communicate information to this patient about diagnosis, prognosis, treatment options etc.?
4. Ask about value of a decision-aid for GPs re shoulder pain, and what information might be useful, e.g. in relation to either prognostic or diagnostic information.
5. What advice did you give to the patient about self-management, work and other activities, and what influenced this advice?
   - Did you use any resources to assist in giving advice, e.g. a specific leaflet or direction to NHS website etc.?
6. What are your views about establishing an effective therapeutic alliance?
   - How did you try to achieve this in the specific consultation?
   - Importance of continuity and having an established relationship with the patient?
   - Did the extent to which you were able to establish this alliance influence your decision-making (e.g. diagnosis, estimating prognosis, referral for diagnostic tests, treatment choices)?
   - How confident do you feel that this patient will have followed your advice?

# Close of discussion

- 1. Any other final remarks/additional views.
  2. Check that consent is still in place.
  3. Reimbursement of travel expenses etc. (where appropriate).
