# Supplementary material for: Patients’ and clinicians’ perspectives towards primary care consultations for shoulder pain: qualitative findings from the Prognostic and Diagnostic Assessment of the Shoulder (PANDA-S) programme
Source: BMC Musculoskelet Disord. 2023 Jan 2;24:1. doi: 10.1186/s12891-022-06059-1 (PMC9805906; doi:10.1186/s12891-022-06059-1)
Supplement: Supplementary file 3 — Supplementary file C. PANDA-S Interview Topic Guide: Clinicians - Physiotherapists. [file 12891_2022_6059_MOESM3_ESM.docx]

PANDA-S Interview Topic Guide: Clinicians -Physiotherapists

# Introduction

- 1. Check that participant has read and understood the PIS.
  2. Explain arrangements for: consent, recording, anonymity, expenses where appropriate etc.
  3. Check clinician’s recall of specific patient and consultation being discussed (if applicable); if necessary arrange to use consultation notes, patient history as an aid.
  4. Record the qualifications of the physiotherapist (level, experience)

# Views and experiences of treating/managing shoulder pain

1. What factors influence your treatment and referral decisions for shoulder pain patients (first presentation)?
   - Prompt re what factors might lead you to consider injecting or onward specialist referral?
   - Has decision-making re management been impacted by the Covid pandemic?
     - Has this changed how you’ve approached management decisions in any way? If so, how?
2. Differences in management based on patient characteristics:
   - Age – younger vs. older patients?
   - Those in work vs. retired patients?
   - Acute injury vs. longer term pain problems?
3. Mode of consultation – differences in consulting with shoulder pain patients remotely vs. F2F in current Covid context:
   - Suitability of remote consulting for shoulder pain
   - barriers/ challenges
   - what would prompt you to bring a patient in for a F2F consultation?
   - How do you see the use of remote consulting for shoulder pain going forward?
4. How important do you regard making a diagnosis in the case of shoulder pain?
   - How confident do you feel diagnosing shoulder conditions?
   - Prompt re role of physical examination (and how this has been affected by Covid)
   - Are there differences for shoulder pain in terms of making a diagnosis when compared to MSK pain in other body site regions?
5. Value attributed to investigations and imaging?
6. How important do you feel it is to communicate a likely prognosis to the patient?
   - How confident do you feel in communicating a likely prognosis?
   - Are there differences for shoulder pain in terms of assessing prognosis when compared to MSK pain in other body site regions?
7. What are your views on reassurance for shoulder pain patients?
   - Does worry/ anxiety appear to have a big role for shoulder pain patients?
   - Do you routinely explore issues re worry/anxiety related to Covid? Was this an issue for the specific patient?
8. What sorts self-management advice would you usually give to patients?
   - Prompt re resources, e.g. a specific leaflet or direction to NHS website etc.?
9. What are your views about establishing an effective therapeutic alliance?
   - How is this achieved?
   - What impacts can this have on patient outcomes?
   - Does the extent to which you were able to establish this alliance influence your decision-making (e.g. diagnosis, estimating prognosis, referral for diagnostic tests, treatment choices)?

# Close of discussion

- 1. Any other final remarks/additional views.
  2. Check that consent is still in place.
  3. Reimbursement of travel expenses etc. (where appropriate).
