# Supplementary material for: Patients’ and clinicians’ perspectives towards primary care consultations for shoulder pain: qualitative findings from the Prognostic and Diagnostic Assessment of the Shoulder (PANDA-S) programme
Source: BMC Musculoskelet Disord. 2023 Jan 2;24:1. doi: 10.1186/s12891-022-06059-1 (PMC9805906; doi:10.1186/s12891-022-06059-1)
Supplement: Supplementary file 4 — Supplementary file D. Theme 1. [file 12891_2022_6059_MOESM4_ESM.docx]

**Theme 1**

Views towards shared decision making

Preference for initial conservative management

Injection for short-term pain relief

Views towards investigation

Considering patient demographics and history

Patient choice

Patient preference for clinician led decisions

(Lack of) discussion of treatment options

Patient preference for discussing available options

Factors influencing management decisions

**Decision-making and discussion of management options**
