# Supplementary material for: Patients’ and clinicians’ perspectives towards primary care consultations for shoulder pain: qualitative findings from the Prognostic and Diagnostic Assessment of the Shoulder (PANDA-S) programme
Source: BMC Musculoskelet Disord. 2023 Jan 2;24:1. doi: 10.1186/s12891-022-06059-1 (PMC9805906; doi:10.1186/s12891-022-06059-1)
Supplement: Supplementary file 5 — Supplementary file E. Theme 2. [file 12891_2022_6059_MOESM5_ESM.docx]

**Theme 2**

Addressing function can be more important than diagnosis

Challenges of remote examination

Clinicians’ (lack of) confidence in giving a diagnosis

Patients receiving mixed messages

Diagnosis informing treatment decisions

Views towards imaging

Important to patients to understand cause of pain

Diagnostic uncertainty

Role of diagnosis in informing management

**Diagnosis of shoulder conditions**
