# Supplementary material for: Patients’ and clinicians’ perspectives towards primary care consultations for shoulder pain: qualitative findings from the Prognostic and Diagnostic Assessment of the Shoulder (PANDA-S) programme
Source: BMC Musculoskelet Disord. 2023 Jan 2;24:1. doi: 10.1186/s12891-022-06059-1 (PMC9805906; doi:10.1186/s12891-022-06059-1)
Supplement: Supplementary file 6 — Supplementary file F. Theme 3. [file 12891_2022_6059_MOESM6_ESM.docx]

**Theme 3**

Patients felt clinician may be unable to provide prognosis

Clinician caution in providing a specific prognosis

Clinicians reported outlining timeframe for recovery

Managing patient expectations

Patients reported prognosis was not discussed

Acceptance of prognostic uncertainty

Patients’ concerns about pain worsening or recurring

Disparity between patients’ and clinicians’ accounts

Prognostic uncertainty

**Discussion of prognosis**
