# Supplementary material for: Patients’ and clinicians’ perspectives towards primary care consultations for shoulder pain: qualitative findings from the Prognostic and Diagnostic Assessment of the Shoulder (PANDA-S) programme
Source: BMC Musculoskelet Disord. 2023 Jan 2;24:1. doi: 10.1186/s12891-022-06059-1 (PMC9805906; doi:10.1186/s12891-022-06059-1)
Supplement: Supplementary file 7 — Supplementary file G. Theme 4. [file 12891_2022_6059_MOESM7_ESM.docx]

**Theme 4**

Trust in the clinician’s expertise

Attempting to reassure despite diagnostic uncertainty

Patient anxiety that pain indicates something serious

Clinicians did not identify patient concerns about serious pathology

Reassurance related to understanding the cause of pain

Disparity between patients’ and clinicians’ views on reassurance

Reassurance about treatment outcomes where diagnosis is unclear

Patient concerns not communicated to clinician

Lack of reassurance where diagnosis is not given

**Giving and receiving reassurance**
