# Supplementary material for: Patients’ and clinicians’ perspectives towards primary care consultations for shoulder pain: qualitative findings from the Prognostic and Diagnostic Assessment of the Shoulder (PANDA-S) programme
Source: BMC Musculoskelet Disord. 2023 Jan 2;24:1. doi: 10.1186/s12891-022-06059-1 (PMC9805906; doi:10.1186/s12891-022-06059-1)
Supplement: Supplementary file 8 — Supplementary file H. Outline of patient-clinician dyad interviews. [file 12891_2022_6059_MOESM8_ESM.docx]

**Outline of patient-clinician dyad interviews**

| **74-year-old male patient.**  **Duration of current shoulder pain episode prior to consultation: 6-12 weeks** | **Example quotes** | **GP, male, over 30 years practising** | **Example quotes** |
| --- | --- | --- | --- |
| The patient reported that during his consultation, the GP did not give him a diagnosis or indicate what he felt the cause of the pain was. | *He didn’t actually say what he thought it was, he just thought the way to go forward was physiotherapy.* | There was disparity in accounts, as the clinician reported that he suspected the patient had a frozen shoulder, and that he had explained this to the patient in the consultation. | *I suspected adhesive capsulitis when I saw him clinically. I always give explanation to patients that what I think it is, in lay terms, a frozen shoulder. And I sometimes give them a leaflet about it or some advice about it, but because it wasn’t definite I didn’t do that. But I thought it was likely a frozen shoulder and I gave him an idea of what it was about and then I referred him to physio.* |
| The patient expressed the view that his pain was not going to get any better and that he would have to live with it long-term. He felt the pain was likely to be age-related. | *My feeling is that I’ve probably got to live with it. I still don’t understand; my feeling is it’s not going to get any better.* *I’m not in any severe pain, any chronic problem at all and I believe it’s probably age-related.* | In contrast, the clinician reported discussing the likely timeframe for recovery with the patient. | *Int: Did you discuss timeframe?*  *P: Yes, you need to give them an idea of timeframe because otherwise they’re not getting better. This goes for all conditions you assess; it’s part of the management. This is all about consultation skills, but you’ve got to tell them the likelihood, how long it’s going to last before getting better, otherwise they come back and they don’t have faith in your management.* |
| The patient discussed being referred for physiotherapy. He reported that the physiotherapist showed him visually the workings of the shoulder and what was causing the ‘stiffness’ he was experiencing. However, he said no one has explained the cause to him. | *She actually showed me a workings of a shoulder [laughs] you know, with the cartilages and that sort of thing. She says it’s a bone over the top, that’s where the stiffness is. You get to a certain way, it’s getting stiff there, it’s resisting. There’s a slight weakness when I was pushing against this arm…* *All she did, all she said was what it isn’t, not what it is.* | In contrast, the GP reported that one of the reasons for referring the patient was to physiotherapy was to confirm the diagnosis of frozen shoulder. | *So he was referred for physio anyway, I gave him a choice of treatments for it and he wanted to go down the physio route for the physio to confirm if it was adhesive capsulitis. And then obviously the way we managed adhesive capsulitis is that either physio and/or injection.* |
| The patient reported that the main reason he went to the GP was for reassurance that nothing serious was underlying his pain, and reported worrying that his pain was caused by serious pathology. | *All I was going for was reassurance really. As it couldn’t be something developing you know. It’s better to catch it when it’s in its early phases…*  *You start to worry when you hear such things as this motor neurone disease. A person that worked for me, he started getting what we thought was tennis elbow as they used to call it… And eventually three years later he died of motor neurone disease. He was one of my colleagues you see, so the back of your mind you think ‘I don’t know why this started’.* | However, the clinician reported that he did not recall the patient needing much in the way of reassurance about his shoulder pain | *P: if it’s musculoskeletal I give [patients] reassurance that I think it’s to do with the shoulder itself rather than anywhere else*  *Int: Did you get a sense that [this patient] required reassurance?*  *P: No, no I didn’t.* |
| The patient indicated that he felt reassured following the consultation because the GP did not suggest his pain was related to anything serious and did not seem concerned. | *He was not over concerned I don’t think. I think he thinks that physio would sort it out really. I was reassured in the way that I came away thinking it’s not something serious.* | The GP felt that his ongoing relationship with the patient facilitated in providing this reassurance. | *Because patients know me and they trust me, and he’s one of my usual patients, then normally that’s enough to reassure them.* |
|  | | | |
| **67-year-old female patient.**  **Duration of current shoulder pain episode prior to consultation: 6-12 weeks** | **Example quotes** | **GP, female, over 25 years practising** | **Example quotes** |
| The patient reported anxiety in relation to the possible cause of her shoulder pain. | *I couldn’t have a shower or wash my hair. I couldn’t do anything…I started worrying that it was something serious* | The GP recognised that the patient was anxious; however, did not identify that this as being related to her shoulder pain. Instead, she reported that the patient was somebody who was generally anxious. | *P: The trapezius muscles were very tense and she was quite an anxious lady.*  *I: Do you recall if that was over anything in particular or was she concerned that there was something more serious going on?*  *P: I don’t think so. I think she’s generally an anxious lady anyway and she’s got other things that go along with anxiety.* |
| The patient reported having told the GP that she had concerns that the pain was radiating down into her chest. However, she did not feel that these concerns were addressed. | *I also told her that the pain was coming down into my chest wall and I was worrying because it was round the side of my breast there. I was worrying about that as well and thinking, ‘Have I got something really wrong?’ which she didn’t seem interested in. She just didn’t care about that. She just ignored it.* | There was some consistency in the accounts, in that GP reported examining the patient’s chest. However, she did not appear to identify this as particular source of anxiety that was in need of reassurance. | *There was some general muscular tenderness really around on the chest wall and into the axilla.* |
| The patient did not feel the GP had addressed this anxiety or reassured her, and reported feeling dismissed by the GP | *I asked and I said to her, ‘Can I go for an x-ray?’ and she didn’t answer. I then said, ‘What about this pain in my breast? It’s very sore when I touch it.’ I said, ‘Could I go for a mammogram?’ but I’m not due for one till about February next year. She said, ‘No, you can’t go for one of them.’* | Due to the nature of the patient’s pain, the GP did not appear to identify her as having anxiety related to her shoulder pain. | *[Patients have] concerns over the pain but it’s not predominantly an anxiety symptom; not unless it’s bilateral. So this was a unilateral problem. It’s just that with it being so tight up in the neck as well, it’s whether or not she’d had primarily a shoulder joint problem but then obviously, she became very tense on that side as a result.* |
| The patient reported that the GP did not communicate a diagnosis or possible cause of pain to her. However, she reported that the GP was on the phone arranging a physiotherapy referral and mentioned frozen shoulder. As a result of this, the patient assumed this was the cause of her pain, but did not ask the GP about this. | *When she spoke to whoever it was in the surgery, she said, ‘I’ve got a lady here with a frozen shoulder. Can you arrange for physio?’ I just assumed that that’s what it was… I thought, ‘Oh, I must have a frozen shoulder.’ That’s the first time somebody had said. I know I have heard of frozen shoulders.* | The GP’s account showed disparity with that of the patient, in relation to the diagnosis she formed based on her examination. | *I really felt that she may well have had a little bit of shoulder arthritis with the crepitus and I certainly felt that she needed some anti-inflammatories because it wasn’t just a shoulder problem.* |
| The patient reported feeling unclear about the medication she was prescribed for her shoulder pain. | *She gave me some tablets which were naproxen and some antidepressants. I’m not depressed. Well, I could be but they weren’t for depression.* | The GP explained the reason for prescribing Amitriptyline to the patient, but does not report on whether this was discussed with the patient. | *There was also that question about whether or not some of this could have been related to tension and that’s why I gave her some amitriptyline to use at night.* |
|  | | | |
| **60-year-old male patient.**  **Duration of current shoulder pain episode: 6-12 weeks** | **Example quotes** | **Male GP, over 20 years practising** | **Example quotes** |
| The patient reported that the GP did not offer a diagnosis for his shoulder pain, which he attributed to the GP being uncertain about the cause. | *I could understand him not [offering a diagnosis], because he wasn’t sure, it’s better for him to be more sure to give you an indication of what’s wrong.* | In contrast, the GP reported that he had an idea of a possible diagnosis, and he indicated that there was some discussion of this within the consultation. | *When I examined him, he actually had a good range of movement. There was no loss of power and there was no obvious neurology. My thinking was along the lines of either rotator cuff or possible impingement…* *When this chap said, ‘What is rotator cuff syndrome?’ I’ll show a picture on that and say, ‘Look, these are the tendons or joints the way it is. This is what we’re aiming to do.’* |
| The patient reported being referred for an X-ray by his GP, and also being referred for physiotherapy.  He reported being disappointed that the X-ray had come back clear, and said he felt ‘puzzled’ by this. | *Oh he sent me for an X-ray that was it, I’d forgot about that. They sent me for an X-ray at [nearby] Hospital, I thought great, that will show up everything that I need. But it didn’t, it came up, nothing wrong. I hoped they’d see something so I was a little bit puzzled at that* | The GP provided more detail about the X-ray results, but his account showed consistency with the patient’s in concluding that the results did not identify a clear cause of the patient’ pain. However, there appears to be some disparity with the patient’s account, in that the GP indicated that the results ruled out anything ‘terrible going on’. The patient did not perceive that that the results had led to any diagnoses being excluded. | *The long and short of it was that the X-ray showed line of the AC joint; glenoid humeral joint appears well preserved; small inferior osteophyte glenoid; no evidence of calcific tendinopathy. It’s not very helpful but at least nothing terrible is going on.* |
| The patient did not perceive that the X-ray results had led to ruling out any conditions or diagnoses, other than ruling out a previous diagnosis he had been given for pain in his other shoulder. | *I can’t remember us ruling out much, other than ruling out what it was last time because I’d done exactly the same exercises and possibly made it a bit worse. So he was fairly happy to rule out that it was the same sort of problem that I had on my left shoulder. I can’t really think that we went into any other detail as to what it could be.* |  |  |
| Having discussed the X-ray results, the patient still expressed concerns that there may be something serious underlying his pain | *I mean you always think these things, is it some sort of bone cancer or I’m not sure what can be done on ligaments or muscles that sort of would be more serious. I’m not that familiar with things that go wrong in your shoulder. But I mean yes you always sort of wonder could it be something very serious.* | These concerns did not seem to be identified by the GP, who felt that the patient was more focused on resolving his pain as opposed to exhibiting ‘anxiety’ about the pain. | *[The patient] said, ‘I’d like not to have this pain please, if you can?’ rather than anything else. I certainly wouldn’t describe anxiety as a presenting feature with this chap.* |
|  | | | |
| **69-year-old male patient.**  **Duration of current shoulder pain episode: 2-6 weeks** | **Example quotes** | **GP, female, over 30 years practising** | **Example quotes** |
| The patient identified the impact of shoulder pain on his sleep as being the most problematic issue. | *And it was a few days after when it started to occur and I couldn’t sleep. Oh, it was a nightmare. It just ached. The whole of my arm ached and I thought, ‘Ah, I can’t put up with this’.* | The GP also identified this as being the issue that the patient was most concerned about. | *So I think he said to me that he’d been having this problem with shoulder pain, I think it had been particularly affecting his sleep and things…* *It was the sleep disturbance really I think that was the main concern from him.* |
| The patient reported having been referred for an ultrasound scan, the results of which were then discussed with the GP. | *Then I went and had a scan and that’s where she found that it was a torn muscle tendon and there was a part of the joint was inflamed as well.* | The GP, however, gave a different account of the scan results. | *there was bicipital tendinosis and it says that the small effusion in the bicep tendon sheath, possibly due to bicipital tendinosis or secondary to glenohumeral effusion.* |
| The patient reported that, following discussion of the scan results, he was offered a corticosteroid injection. Whilst he reported having been hesitant about an injection, he expressed a strong preference for the decision about his treatment to be led by the GP. He reported that the injection led to an improvement in his pain. | *I couldn’t care less about medical stuff. I’ll leave that to the doctors. I’m not interested. All I want them to do is to treat me. So all I wanted, get rid of this pain in my arm, whatever way, let them tell me.* *I didn’t fancy an injection in my shoulder. I got home and thought, ‘What have I done?’. But do you know, from then on after that night it went easier.* | The GP reported that the reason for deciding on an injection was that the patient indicated that he was looking for a ‘quick fix’ for his pain, particularly due to the impact it was having on his sleep. She reported that she explained to the patient that this option can having varying results in reducing pain. | *So he came back and he said the pain was still the same, it was still disturbing his sleep, what would basically be a quick fix. So we spoke about the pros and cons obviously of injection. And on the [date] he did have an injection…* *I always sort of say to people there’s no guarantees, if it works it often works really well but I can’t tell you for how long it’s going to give you relief, but it might not work at all.* |
| The patient felt that his shoulder pain was related to old age and therefore not likely to resolve or improve in the future. It was unclear, however, whether the prognostic outlook was discussed with the GP. | *I think I accept it’s going to stay with me. It’s one of the things about getting old.* | The GP reported always discussing prognosis with patients with shoulder pain, however, it is unclear whether this was discussed with this patient, who had the impression that his pain would not improve. | *I do always try and sort of give them the expectation that it could well turn into a more chronic problem and therefore ways of managing it* |
|  |  |  |  |
| **38-year-old female patient.**  **Duration of current shoulder pain episode: 6 months- 1 year** | **Example quotes** | **GP, female, over 30 years practising** | **Example quotes** |
| The patient was referred by the GP for an ultrasound scan, which indicated a diagnosis of bursitis; however, the patient referred to this as ‘galitis’. She reported being prescribed anti-inflammatory medication, and said she felt reassured having received a diagnosis and knowing that something could be done to address the pain. | *My GP did send me for an ultrasound, and that came back with oh let me see if I can get the name of it right… galitis? the doctor told me I’d got that and basically just to take anti-inflammatories…I was quite relieved actually to know that there is actually something wrong with it and not just “oh we don’t know what it is you’ll have to put up with it”* | There was consistency with the GP’s account, who reported that she had formed a working diagnosis of bursitis, which was confirmed by the ultrasound scan. | *I think clinically I thought it was a bursitis because of where she was tender and how she’d described it and that she couldn’t lie on that side in bed and so on and so forth. So I had put a query bursitis. And it came back showing yeah mild thickening of the subacromial bursa suggesting a degree of bursitis and some ACJ minor degenerative changes.* |
| However, the patient was unclear about what other treatment options might be available moving forward, and expressed concerns about taking anti-inflammatory medication in the longer-term. | *With not really knowing what I can take and what can be done for it, like physio-wise or treatment-wise or like I say I don’t want to just keep taking anti-inflammatories all the time because eventually your body gets used to taking certain things and eventually it wouldn’t work for me anyway.* | The patient’s concerns about longer-term management did not appear to be identified by the GP, though it was not clear if this was communicated by the patient. The GP talked about the possibility of a corticosteroid injection if the pain is still impacting on the patient. | *I think in my mind what I would normally do is get her back, reassess her clinically, you know, if she’s still got the pain, particularly with that sort of mild bursal thickening, in terms of a quicker fix, [I’d consider] did she want an injection.* |
|  | | | |
| **74-year-old male patient.**  **Duration of current shoulder pain episode: over 1 year.** |  | **GP, Female, 3 years practising** |  |
| The patient reported that he was not given a diagnosis for his pain and was just told it was due to ‘wear and tear’.  He reported having been referred for an X-ray, but says the results had not been shared with him by the GP, and was unclear whether the GP had seen the results. He reported being referred for an ultrasound scan which he was waiting for at the time of the interview, and had also been referred for physiotherapy. | *P: It’s in that wear and tear over the years, you know. I had a physical, manual job and that’s probably the result of it, yeah.*  *Int: [The GP] said it’s wear and tear?*  *P: Yes. Wear and tear, yeah.*  *I: Did she discuss the results of the X-ray with you or…?*  *P: No, not as such…I’m not sure whether she said she’d seen the X-ray or not, I can’t remember.*  *I: Okay. So you’ve actually not had the results given to you then?*  *P: No.* | Whilst the GP’s account showed some consistency with the patient’s, there was also disparity in that the GP reported discussing the results of the X-ray with the patient, and explaining the reasons for referring him for an ultrasound scan. |  |
|  |  |  | *We talked about it briefly and said ‘Look, the X-ray isn’t showing us a huge amount’, from what I remember, and I quickly re-examined him and just said ‘Look, I think you probably need an ultrasound to have a look at the soft tissue around your shoulder and check that the muscles are intact and the ligaments and things are intact’ and he said ‘Yes, that’s fine’.* |
| The patient reported that the GP did not want to commit to giving him advice about managing the pain until he had seen the physiotherapist | *I asked [the GP] whether, I do regularly exercise, whether I could carry on with it or whether I should rest it and she said we’ll see what the scan reveals…* *she didn’t really want to commit to whether it was better to have exercises or rest it. She’d be advised by the physiotherapist on that one.* | Again there was some disparity with the GP’s account, in that she reported advising the patient to keep his shoulder moving, but to avoid strenuous activity. | *[I said] try and keep moving. With any kind of joint pain, I generally say try and keep moving, gentle exercise…try and avoid heavy lifting and sort of anything that’s putting any sort of physical strain like real pushing or pulling exercise, nothing like that. But you know, keep the shoulder moving, make sure that you’re not sitting there holding it.* |
|  | | | |
| **53-year-old male patient.**  **Duration of current shoulder pain episode: 6 months – 1 year** |  | **First Contact Practitioner (FCP) – Advanced Practice Physiotherapist, Female, over 15 years practising** |  |
| The patient highlighted that the impact of his shoulder pain on his sleep was what most concerned him. He also highlighted other broader factors that he felt contributed to this, but it is not clear if he discussed this with the FCP. | *It was sleeping. My sleep was getting worse and worse. Well there’s a couple of contributing factors in that. Basically the Covid situation, mental health, just being bloody bored to be honest. So that might have something to do with it, but it seems to have become a bigger issue, maybe because I was focusing on it more, I’m not sure* | There was some agreement in the accounts, as the GP highlighted the impact of pain on the patient’s sleep and reported requesting analgesia to help with this. | *He asked me about medication because he said it was affecting his sleep at night time* *and he actually approached me about amitriptyline and so I sent through a request through to the GP for that. it just about trying to get their sleep better because that has a huge impact on life in general if you're not sleeping very well, doesn't it?* |
| The patient had previously been diagnosed as having subscapularis tendinitis, and this same diagnosis was confirmed by the clinician. However, the patient was dissatisfied with the examination that was carried out via video call, and as a result was not convinced of this diagnosis. He expressed a strong preference for in-person examination. | *I was disappointed really that she was taking my diagnosis without even sort of questioning the whole, well examining me really. I’d have expected an examination of sorts to check it is the same thing. I know it’s the same thing, but I’m a layman, I couldn’t diagnose anything. Whereas as the professional, I feel she should’ve called me in and said, ‘Right, let’s have a look. Is it this?’ and that’s that.*  *I’ve had a video call from her and it was comical, absolutely comical. ‘Right put your phone in front of you, lift your arm up so I can see it’. Come on. It was absolutely comical. ‘Look, if I put my phone there I can’t see it, so I can’t see what you can see, so I can’t do the exercise’. It was nice to try it, but absolutely waste of time.* | There was disparity in the FCP’s account. She reported confidence in her diagnosis of tendonitis in line with patient’s previous diagnosis, and had not felt there was a need to invite the patient to attend in person. | *Yeah, I managed to do a video call and what I noticed was that he had restriction into flexion of 160o and pain on abduction of 110o. He had some restriction in taking his hand behind his back, he could reach to around about L5 and his horizontal flexion was about three quarters range. I looked at his cuff and so I did the Gerber's Lift Off Test which did increase his pain and it was noticeably weaker but he did still lift his hand off his back, so that suggested to me that was in keeping with the subscapularis tendinopathy that they'd found on the ultrasound.* |
|  |  |  |  |
| The patient did not feel that the impact of his pain on sleep had been fully addressed within the consultation. | *I: Did she ask you about the impact that the pain was having on your everyday life and on your sleep and those sorts of things?*  *P: Sort of glossed over it. It wasn’t a structured sort of, ‘Right, are you losing sleep? How do you feel about this?’ It was just a general, ‘How do you feel? Why did you put in to speak to us?’* | Again there was some disparity with the FCP’s account. Whilst she did not discuss the advice she had given this specific patient, she talked about sleep as being something that was regularly addressed in shoulder pain consultations. | *It's about advising them on sleeping positions. Quite often, sleeping with their sore arm hugging a pillow, just so that it's slightly abducted away from their body, can offer them a little bit of relief at night time. Other things that I'd talk to them about would be taking perhaps some medication before they go to bed and using heat on the shoulder which could be quite soothing. If they really don't get on with the heat, then their alternative is the ice.* |
|  | | | |
| **65-year-old male patient.**  **Duration of current shoulder pain episode: over 1 year.** |  | **First Contact Practitioner (FCP) – Advanced Practice Physiotherapist, Female, over 15 years practising** |  |
| The patient reported that his shoulder pain was significantly impacting on his sleep. | *The pain, I just wasn’t sleeping. It was waking me up every single night of the week. And gradually, not a lot, but it was gradually getting worse each sort of week as time went by.* | This was recognised by the FCP, who identified this as the main reason for the patient consulting. | *I think the reason perhaps for him presenting more so at the moment is that it was starting to wake him in the night. As I say, he had had some physio in June time of last year but he was finding that the night time symptoms were what was starting to bring him back.* |
| The patient reported feelings of uncertainty about the cause of his shoulder pain, having been told by his GP that he had arthritis, but by another professional that he had subacromial shoulder pain (i.e. impingement). | *Well they said arthritis and then they sent me up to the hospital to have an X-ray and a scan. All I’ve had is phone calls from them, which is what everybody else is getting. You know, it’s just the appointment is on the phone. And each time they’ve told me something different about the X-rays or about the scan. Yeah, it’s different things going on, they’ve been telling me different things…The man who did my scan, he told me it was quite a bad impingement. And then the doctor told me it was arthritis, so I sort of didn’t know which one to believe to be honest.* | The FCP reported that, based on the patient’s X-ray and her examination, both diagnoses were indicated, though she does not explain how these diagnoses were communicated to the patient. | *He had an X-ray of his shoulder which showed calcific rotator cuff tendonitis and subacromial bursitis and then the ultrasound showed some moderate OA (osteoarthritis) changes in his acromioclavicular joint. Normal long head of biceps, no rotator cuff tear but some thickening of the bursa which was felt likely to be representative of impingement.* |
| The patient reported feelings of uncertainty about his care pathway and the treatments available to him. He said he felt that the co-codamol prescribed for his pain was not appropriate for him. Whilst he noted that the possibility of having a corticosteroid injection had been discussed, he reported that this has not been followed up on, and that he was told this was not an option at that point in time due to Covid-19 restrictions. | *I’m completely left in limbo. I mean the last doctor, the physio service asked him to give me a ring about some painkillers. I just said I would like to have a good night’s sleep every now and again. The doctor phoned me and all he gave me was co-codamol for the pain. I mean I’m a driver by trade, I’m delivering drugs for other people and it’s got on it “don’t take if driving”. So I can’t see any point in it really.*  *I mean they said I could have the injection, the steroid injection. Two people have mentioned that, but they’ve never ever come back to tell me about it because they can’t do it while the Covid’s on apparently.* | The FCP’s account showed some disparity, in that she outlined a clear treatment pathway for the patient. She reported that her impression was that an injection was not the patient’s preference, as opposed to this not being available to him. She also reported having confidence in the patient’s diagnosis on the basis of her visual examination, the X-ray findings, and an ultrasound that he had had in the past, which is in contrast with the uncertainty and confusion expressed by the patient. | *As his symptoms were ongoing, I chose to refer him for further physio so that they could obviously see where he was at from the exercise point of view and also consider something like a steroid injection, either into the subacromial space or into the acromioclavicular joint, dependent on where he was most symptomatic. Although, I got the impression he wasn't overly keen for that to be the preferred thing that he tried. Again, I think, at that point in time, rather than bring him in, based on the X-ray and the ultrasound that he'd had in the past and looking at his range of movements, I felt happy that things were in keeping with those investigation findings and that, as I say, further physio and possibly the injection was what could be considered next for him.* |
|  | | | |
| **39-year-old female patient.**  **Duration of current shoulder pain episode: over 1 year.** |  | **GP, female, over 20 years practising** |  |
| The patient reported that the GP had given an explanation about the possible cause of pain, but seemed unclear about the cause. She felt that there was uncertainty from the GP, suggesting the GP was ‘guessing’. | *She thought that potentially, something to do with the ligaments, just something’s worn out there and she thinks the bones are actually scratching on each other and that’s why it’s causing this sharp pain. It’s like the nerve’s been damaged there, that’s what she thinks has been going on. She can’t say 100% but that’s what she's guessing.* | The GP’s account showed some disparity, as she indicated making a probable diagnosis, which did not appear to correlate with what the patient picked up from the discussion. The GP also indicated a greater degree of confidence in her diagnosis than was interpreted by the patient. | *So I thought that she probably had tendonitis. So she didn’t have frozen shoulder, she didn’t have any kind of bony issue with her shoulder. If I recall I thought she might have supraspinatus tendinitis, if I was being precise…So yes, I thought she had a tendinitis, almost certainly of the shoulder.* |
| Whilst the patient appeared unclear about the cause of her shoulder pain, she did report feeling reassured that there was nothing serious causing the pain. However, she reported avoiding movements that would cause pain due to concerns about how it may impact her shoulder. | *At least I know it’s nothing, you know like serious, but then knowing that you know the bones, the joints are scratching, and potentially if there's any liquid she said because the way the bones are moving on one side could be too much liquid and the pressure causing the pain as well. So, it really depends like trying to, you know, avoid doing movements that will hurt me but again, you know I can’t do it all the time so the pain is there.* | The GP reported that enabling patients to understand the cause of pain can lead to reassurance. However, she reported encouraging patients to move ‘within the limits’ of pain, which appears to contrast with the patient’s perspective that she should avoid movements that cause pain. | *I: How important do you feel that is in a GP consultation to try and give the patient and indication of a possible cause if you’re able to? Do you see that as important for your role?*  *P: I think it’s really important in lots of different ways. So, I don’t think it’s so important actually for me because actually for the treatment of shoulders there’s a lot of similarities between lots of different conditions and lots of treatments. But I think for patients, if they have confidence that we know what we’re dealing with I think it makes a massive difference.*  *So for all sorts of musculoskeletal things I will try and reassure people that pain does not mean something awful is going on. Actually moving within the limits of pain is important because joints getting stiff is a problem. So I’ll usually have some sort of encouragement to at least try and keep things moving.* |
| The patient saw value in having an in-person examination of her shoulder, which she appeared to see as key to identifying the cause of her pain. | *It was definitely worth it when she examined, even you know in Covid, she was happy to like go and touch the shoulder, lift my arm and all that so it was actually proper examination. It was nice to be not just over the phone when she had no idea what's going on, but yeah, that was good.* | The GP’s account correlated with the patient’s, in recognising the value of in-person examination from a patient perspective. She suggested that this can give the patient confidence that the GP is able to identify the cause of their pain. | *I do think examining a shoulder makes a difference. And not always as I said before because it helps me, but because it helps the patient understand that I do really know what I think is wrong. So I do think examination is an important part of assessing a patient in all sorts of ways. So I think losing that is a challenge because I want the patient to have confidence that I do know what the right thing is to do for their shoulder and I understand that if they don’t think I’ve looked at it they quite rightly think, ‘How on earth does she know what’s wrong with my shoulder?’ So I kind of think that missing an examination is tricky. And actually so she’s quite a good example.* |
